# Is serum AMH a suitable biomarker to determine litter size or fetal sex in goats?: A preliminary study

**DOI:** 10.1007/s11250-025-04315-9

**Published:** 2025-02-13

**Authors:** Ece Koldaş Ürer, Ayşe Merve Köse, İlknur Pir Yağcı, Mert Pekcan, Ahmet Gözer, Onur Bahan

**Affiliations:** 1 Faculty of Veterinary Medicine, Department of Obstetrics and Gynaecology, Hatay Mustafa Kemal University, Hatay, Turkey; 2 Faculty of Veterinary Medicine, Department of Obstetrics and Gynaecology, Kırıkkale University, Kırıkkale, Turkey; 3 Faculty of Veterinary Medicine, Department of Biochemistry, Ankara University, Ankara, Turkey; 4 Faculty of Veterinary Medicine, Department of Obstetrics and Gynaecology, Yozgat Bozok University, Yozgat, Turkey

**Keywords:** AMH, Goat, Fetal sex, Pregnancy

## Abstract

This study was conducted in Damascus goats to i) investigate the relationship between serum AMH concentrations and litter size, ii) evaluate the change in serum AMH concentration between the 35th and 135th days of pregnancy, iii) determine whether fetal sex affected the maternal AMH level. Estrus was synchronized in 110 goats (intravaginal sponge containing medroxyprogesterone acetate for nine days, combined with d-cloprostenol and Pregnant Mare Serum Gonadotropin at sponge removal). Blood samples were taken on the 4th and 9th days of synchronization. Goats at estrus were mated naturally. Study groups were established based on the number of fetuses on ultrasound examination as Group I (*n* = 18, goats carrying one fetus) and Group II (*n* = 18, goats carrying multiple fetuses) (Experiment I). Additionally, blood samples were taken every 20 days between the 35th and 135th days of pregnancy. After the parturition, groups were formed according to the sex of the kids; single/multiple male fetuses (GroupM, *n* = 8), single/multiple female fetuses (GroupF, *n* = 8), and fetuses of the opposite sex (GroupFM, *n* = 8), (Experiment II). There was no significant relationship between serum AMH concentration and litter size (*P* > 0.05). As pregnancy progressed maternal AMH concentration tended to decrease (*P* < 0.001), and fetal sex did not affect maternal AMH (*P* > 0.05). In conclution circulating AMH was not an ideally biomarker for determining litter size on natural breeding or predicting fetal sex in goats. Futher studies with larger sample sizes and the reduced individual variables (age and ovarian activity) are recommened.

## Introduction

The Anti-Müllerian Hormone (AMH) is a homodimeric glycoprotein belonging to the transforming growth factor β (TGF-β) family with essential roles in cell reproduction, differentiation, and apoptosis (Gautam et al. 2021). The role of the male fetus in sex differentiation was first described in the 1940s through the inhibition of the Müllerian duct (Jost et al. 1973; Massagué et al. 1994; Josso et al. 2006). It was discovered in subsequent studies that it not only played a role in male sex differentiation but also in the development and functioning of female reproductive organs (Teixeira et al. 2001; Josso and Clemente 2003). AMH is known to prevent early depletion of the follicular reserve by preventing a certain amount of primordial follicles in the ovaries from migrating to the growing follicle pool (Durlinger et al. 2002; Gigli et al. 2005). In goats, as in many other mammals, AMH inhibits the activation of primordial follicles (Rocha et al. 2016). AMH also plays a crucial role in female reproduction by regulating follicular development and decreasing the Follicle stimulating Hormone (FSH) responsiveness of preantral and small antral follicles (Dewailly et al. 2014).

During the Multiple Ovulation Embryo Transfer (MOET) program, a positive correlation was obtained between the plasma AMH concentration before exogenous gonadotropin injection and the number of oocytes and embryos produced after stimulation (Mossa et al. 2007; Guerreiro et al. 2014; Lahoz et al. 2014). Furthermore, viable offspring rates were higher in pregnancies following the transfer of embryos collected from donors with higher serum AMH concentrations (Ghanem et al. 2016). Recent studies on goats have focused on the role and availability of AMH in superovulation and embryo transfer, as thet are in many other ruminants (Soquila and Mingala 2017; Karakas Alkan et al. 2020). Monniaux et al. (2011) claimed that a single AMH concentration measurement in goats whether in or out of breeding season may be a suitable marker for assessing fertility. Few studies investigating the relationship between circulating AMH levels and pregnancy outcomes have reported conflicting results. In dairy cows, reduced levels of circulating AMH were associated with reduced pregnancy rates (Jimenez-Krassel et al. 2015). Likewise, cows with higher circulating AMH concentrations eight days after insemination had greater conception rates (Ribeiro et al. 2014).

The predictability of fetal sex based on maternal AMH was investigated due to the role of AMH in sex differentiation. Severeal studies investigated maternal AMH concentration as an early fetal sex marker in human pregnancies; however, conflicting results have been reported (Empey et al. 2012; Köninger et al. 2013; La Marca et al. 2005; Nelson et al. 2010; Pankhurst et al. 2016). A recent animal study in cows reported that maternal AMH was affected by the fetus in the male sex. Accordingly, plasma AMH concentrations of cows carrying male fetuses between 53–135 days of pregnancy were significantly higher compared to the cows carrying female fetuses (Stojsin-Carter et al. 2017). Nevertheless, this possible relationship has not yet been revealed in goats with high probability of multiple ovulations and bearing multiple fetuses of both the same and the opposite sexes. Accordingly, this study aimed to elucidate three points in a goat herd that received estrus synchronization protocol during the breeding season i) determining of litter size based on AMH concentration measurement during during estrus synchronization protocol in adult female goats, ii) revealing the level of maternal AMH in goats during pregnancy, and ii) determining the possible change in maternal AMH level according to fetal sex.

## Materials and methods

This study was conducted in the Antakya district of Hatay, Türkiye (36.42° N latitude and 36.23° East North longitude) from July 2021 to February 2022 on a commercial dairy goat farm, and examined 110 multiparous Damascus goats aged 3 to 6, weighing 40 and 60 kg, which had undergone estrus synchronization, to determine the goats to be included in the study groups. Goats were housed in free stables. The goats were kept in free stables. The goats were fed ad libutum on pasture for 14 h a day until November. From November to February, the goats were fed ad libitum with grass hay and concentrate in the stable. They had free access to water and mineral blocks during the study period.

### Design of the experiment

In the study, two experiments were designed on different goats from the same herd. Accordingly, progesterone-based estrus synchronization was administered to 110 female goats in the herd.

### Estrus synchronization

At the beginning of the breeding season (Late July), intravaginal sponges containing medroxyprogesterone acetate were placed into the vaginas of goats under appropriate aseptic conditions (60 mg of MAP, Esponjavet, Hipra, Spain). Sponges were kept in the vagina for nine days, and 0.075 mg d-cloprostenol (Senkrodin ®, Vetaş, Türkiye) along with 400 IU PMSG (PMSG-Intervet®, Türkiye) were administered intramuscularly to all goats at sponge removal. The 4th day, which corresponded to the middle of the synchronization program, and the 9th day, which was the day of sponge removal were selected as the key days for AMH measurement, and blood samples were obtained from the entire herd for further evaluation. After synchronization, the goats in estrus were mated with five fertile bucks (goat:buck ration 22:1). Bucks joined the flock for three days, twice a day, one hour in the morning and evening, starting twelve hours after the removal of the sponges. Mated female goats were placed in a separate compartment of the barn.

- Experiment 1: On the 35th day after mating, a transrectal pregnancy examination was performed on the synchronized herd using a real-time ultrasound device with a 6–8 MHz probe (Falco 100, Pie Medical, Netherlands). Transabdominal ultrasonographic examination was repeated on the 55th day of mating to confirm pregnancy and eliminate the possibility of early embryonic death. The number of fetuses carried by the pregnant goats was noted. In addition, the birth records of kids were compared with the results noted in the ultrasonographic pregnancy examination. The goats, which showed the same data in both notes, were included in the study group of Experiment 1. Accordingly, the study groups of Experiment 1 were determined as Group I (*n* = 18, goats carrying one fetus) and Group II (*n* = 18, goats carrying multiple fetuses). The goats were divided into groups using the random sampling method. The AHM levels in the blood samples obtained from 36 goats on the 4th (S4) and 9th (S9) day were measured, and the differences between the groups were statistically analyzed.
- Experiment 2: A total of 6 blood samples (P35, P55, P75, P95, P115, and P 135) were obtained from all goats whose pregnancy was diagnosed after synchronization, starting from the 35th day after mating (35th day of pregnancy) and every 20 days. The parturition of the goats was monitored, and the sexes of the newborn kids were noted. The study groups of Experiment II were formed retrospectively, considering only eight goats carrying female fetuses according to the sex records of the newborn kids. Accordingly, 24 female goats were used as follows; goats carrying one male fetus or multiple male fetuses (GroupM, *n* = 8), goats carrying fetuses of the opposite sex (GroupFM, *n* = 8), and goats carrying one female fetus or multiple female fetuses (GroupF, *n* = 8). The AMH results were evaluated statistically.

The goats from which blood samples were used in Experiment 1 were not included in Experiment 2.

### Collection, storage, and analyses of blood samples

Using a 21 gauge blood collection needle, 8 ml of blood samples were taken from the goats on S4, S9, and throughout pregnancy (P35, P55, P75, P95, P115, and P135) from the jugular vein into anticoagulant-free tubes. The blood samples were centrifuged at 3000 rpm for 10 min, and the serums were extracted. Serums were transferred to eppendorf tubes and stored in a deep freezer at −20 ºC until analysis.

The commercially enzyme-linked immunosorbent assay kit (Goat Anti-Mullerian hormone ELISA Kit, Cat No.MBS267219) was used for determination of serum AMH levels according to the manufacturer instructions. The ELISA kit employs the “Double Antibody Sandwich” technique. Primary antibodies were coated in 96 well plates and all individual reagents and samples or standards are incubated one at a time. Extensive washing cycles were performed after each incubation period. Following final wash plates were incubated with Color Reagent before addition of the stop solution. The dual-wavelength absorbance measurements were taken at 450 and 620 nm.

### Statistical analysis

The sample size was calculated with G*Power software version 3.1.9.2 before the selection of groups in both experiments. Results of the sample size calculation showed that the minimum number of goats were 34 and 21 for Experiment 1 and Experiment 2, respectively, considering an effect size of 0.25, an alpha value of 0.05, and a power of 0.80. All statistical analyses of the study were performed on the SPSS 23.1.0 package software. The relationship between AMH levels in the serum of pregnant goats and fetal sex, and the relationship between AMH levels during synchronization and litter size were evaluated by ANOVA. Results were reported as percentages or means ± standard error of the mean (SEM). Statistical significance was defined as *P* < 0.05.

## Results

Among the synchronized goats, two were excluded from the study due to disease and sudden accidental death. In the study, 81 goats got pregnant with the synchronization program, and the pregnancy rate of the herd was 73.6%. None of the goats that were pregnant on the 35th day of the study were determined to have lost pregnancy by the 55th day (embryonic death).

**Fig. 1:**
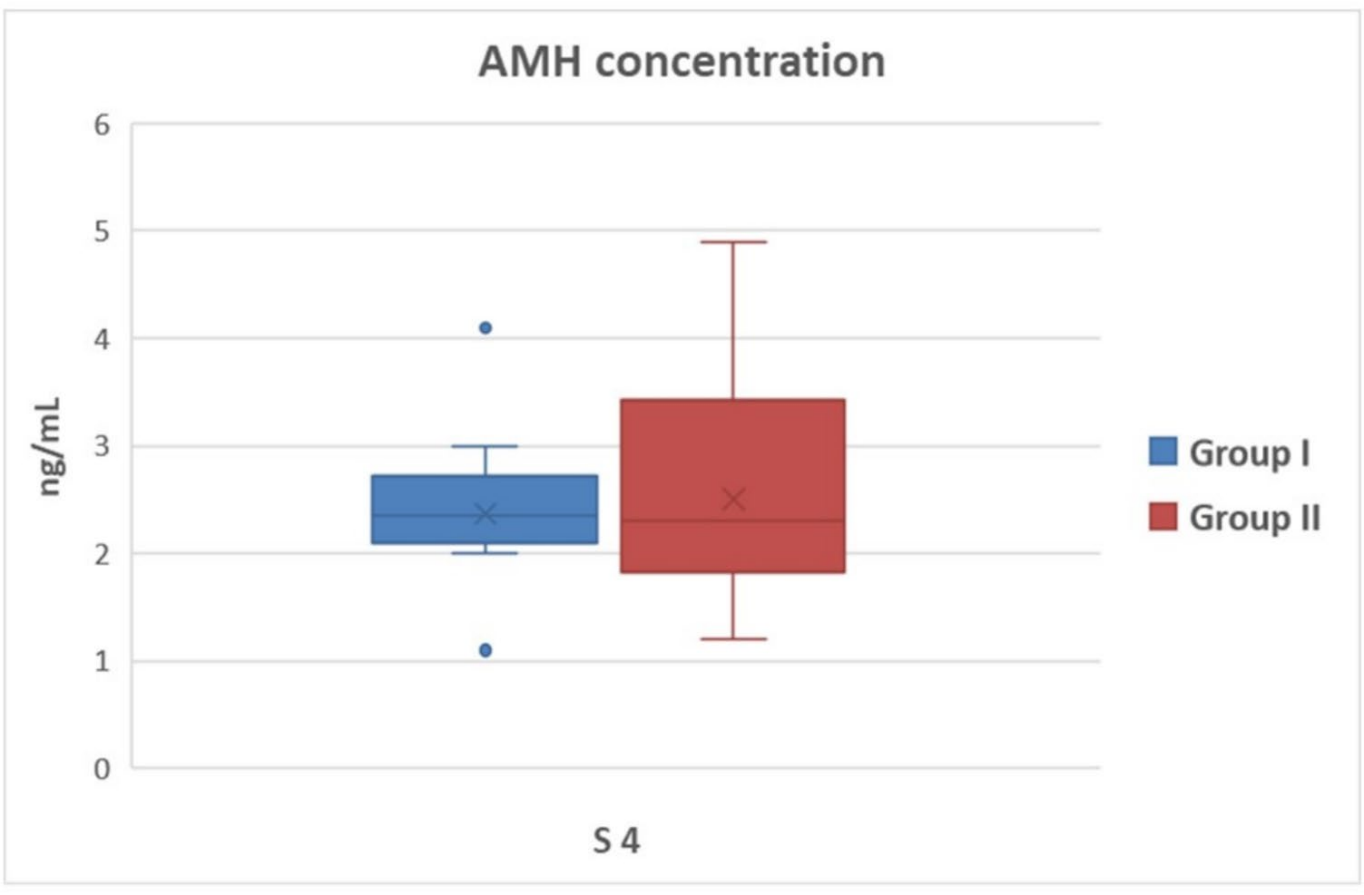
Serum AMH concentrations of goats pregnant with single and multiple fetuses on the 4th day of estrus synchronization. (Group I = goats carrying single fetus, Group II =  goats carrying multiple fetuses, S4: 4th day of estrus synchronization)

**Fig. 2:**
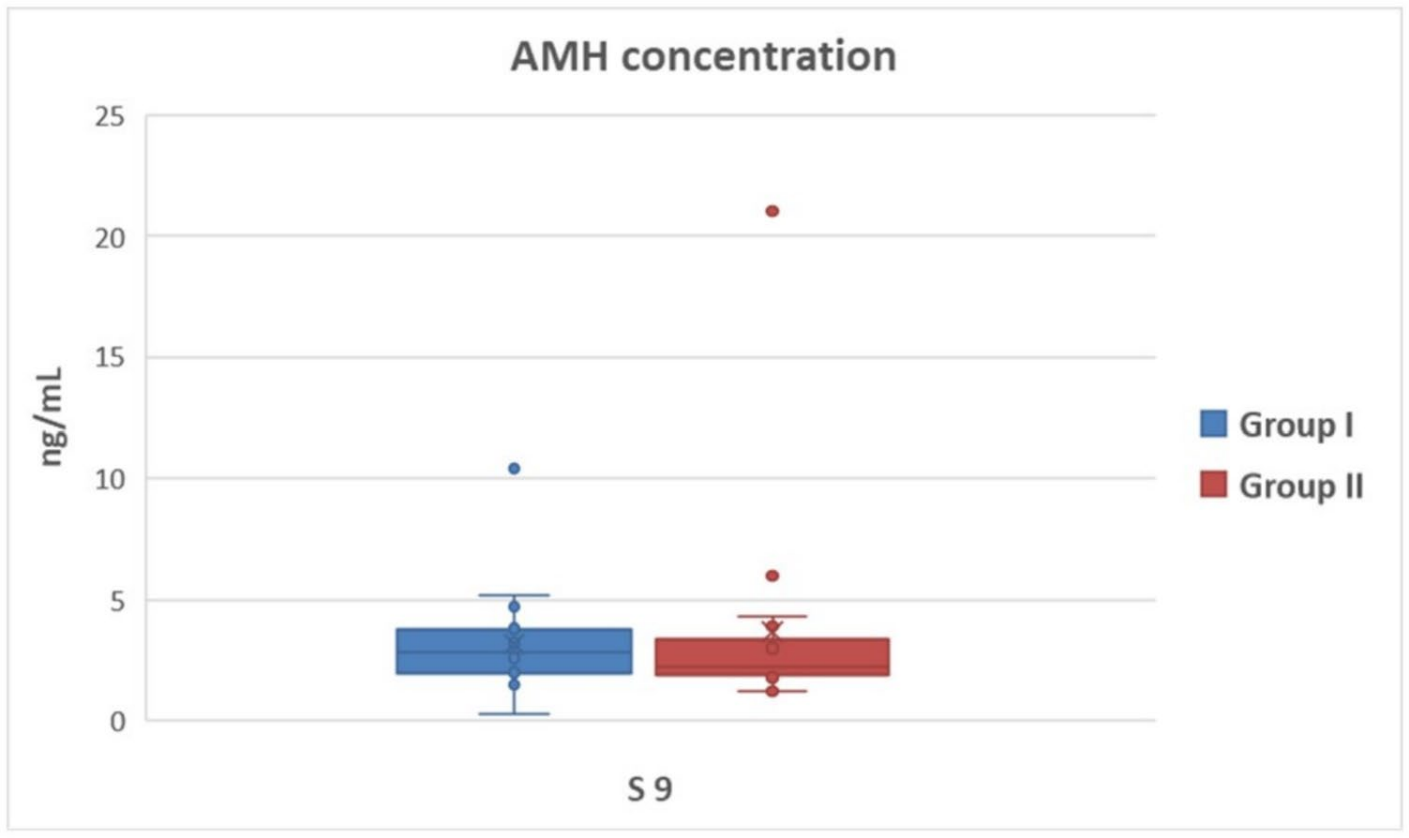
Serum AMH concentrations of goats pregnant with single and multiple fetuses on the 9th day of estrus synchronization. (Group I = goats carrying single fetus, Group II = goats carrying multiple fetuses, S9: 9th day of estrus synchronization)

The AMH concentration in S4 was 2.37 ± 0.16 and 2.51 ± 0.23 ng/ml (*P* > 0.05) in Group I and Group II, respectively (Fig. 1), and the AMH concentration in S9 was 3.18 ± 0.51 and 3.66 ± 1.06 ng/ml (*P* > 0.05) in Group I and Group II, respectively (Fig. 2). In both groups, AMH concentration tended to increase between the two sampling days; however, it was not statistically significant (*P* > 0.05).

**Fig. 3:**
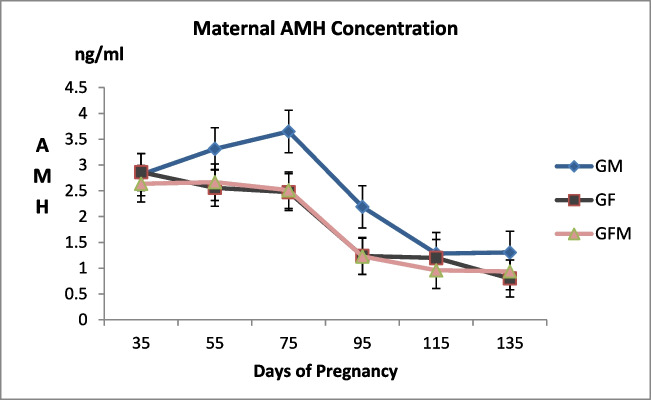
Maternal AMH change by fetal sex during pregnancy. (GM: Group male fetuses, GF: Group female fetuses)

Maternal AMH levels in the serum of pregnant goats monitored in Experiment 2 were determined to be unaffected by fetal sex at any time during pregnancy (Fig. 3, *P* > 0.05). In addition, maternal AMH variation was found to be significantly different in each group during pregnancy (Table 1, *P* < 0.001).

**Table 1:** Maternal AMH concentrations in the groups during pregnancy

	Maternal serum AMH concentration (ng/ml) Mean ± SE						*P*		
							Day	Group	Day x Group
	P35	P55	P75	P95	P115	P135			
Group M	2,82 ± 0,56	3,31 ± 0,68	3,65 ± 0,60	2,19 ± 0,37	1,28 ± 0,34	1,31 ± 0,43	< 0,001	0,325	0,762
Group F	2,86 ± 0,60	2,56 ± 0,36	2,48 ± 0,35	1,24 ± 0,43	1,20 ± 0,41	0,80 ± 0,21			
Group FM	2,64 ± 0,56	2,67 ± 0,53	2,51 ± 0,24	1,23 ± 0,20	0,96 ± 0,33	0,94 ± 0,29			

GroupM: single/multiple male fetuses, GroupF: single/multiple female fetuses, GroupFM: fetuses of the opposite sex

## Discussion

This study determined that the AMH concentration measured on the day of estrus synchronization in adult goats was insufficient to indicate the litter size in pregnancies resulting from natural mating. In addition, it revealed that maternal AMH concentration in pregnant goats decreased as the pregnancy progressed; however, it did not change significantly enough to indicate the fetal male sex.

MOET studies determined the possible response of AMH concentration to superovulation treatment with FSH. It was reported that plasma AMH concentration before FSH injection, corresponding to the 9th day of intravaginal sponge administration in goats, was highly correlated with the total CL and the number of collected, transferred, and frozen embryos (Monniaux et al. 2011). Karakas Alkan et al. (2020), on the other hand, found a significant positive correlation between the circulating AMH concentration at the onset of the synchronization protocol and the total number of CL, total oocytes/embryos, transferable embryos, and excellent quality embryos in Angora goats. Accordingly, in this study it was aimed to determine the relationship between serum AMH concentration and fetus number (single or multiple) in a Damascus goat herd that received estrus synchronization treatment with PMSG, on specific days during sponge administration when the uniformity was expected to be the highest. PMSG administration at the time of lutealitic drug injection during the breeding season enhances ovulation rates, resulting in an increased number of offspring. PMSG has both FSH and LH-like characteristics. For superovulation response in goats, dosages of up to 1000 IU of PMSG are commonly required (Amoah and Gelaye 1990). However, PMSG has an effect on the steroid hormone pattern due to its lengthy half-life, resulting in premature regression of the corpus luteum (Senthilkumar et al. 1998), as well as an increase in the number of persistent large follicles that failed to ovulate, resulting in diminished ovarian response (D'Alessandro et al. 1996; Armstrong et al. 1983). It also stimulates follicular steroid release, which inhibits sperm and gamete transit, oocyte maturation, and early preimplantation embryo development (Gonzalez et al. 2001). Therefore, dosages of up to 500 IU are recommended for fixed-time artificial insemination protocol (Baldassarre and Karatzas 2004). It was expected in this investigation that PMSG delivered at a dose of 400 IU at the time of sponge removal as part of the synchronization protocol would produce an identical response to FSH injections in MOET studies. Nevertheless, no relationship was determined between litter size and AMH concentration on both the 4th (Fig. 1) and 9th days (Fig. 2) of sponge administration (*P* > 0.05). Because PMSG has a lengthy biological activity and can delay the preovulator LH wave for up to an hour, it may have constantly encouraged the production of antral follicles, leading to substantial number of non-ovulated follicles in this study, as previously documented (Hervé et al. 2004). Multiple ovulations may not have occurred due to the reported effect, despite higher AMH concentrations indicating antral follicles and multiple ovulation capacity. On the other hand, the male factor was accepted as a fixed variable in this study. Considering that pregnancy likelihood is directly affected by male fertility as well as female fertility, the male effect could be the second significant reason for the inability to establish a relationship between litter size and circulating AMH concentration. Contrary to the MOET studies, multiple implantations may not have occurred in some goats despite the high potential for multiple ovulations. It was assumed that many variables could have affected the serum AMH concentration. For instance, it was mentioned that the AMH concentration in circulation decreased with age due to the reduced follicle pool in women at advanced ages (Van Rooij et al. 2005; Meczekalski et al. 2016). In goats, the effect of age on circulating AMH concentration was examined in a limited number of studies, and it was stated that AMH concentration started to decrease after the age of 5 (Haldar et al. 2019). It was assumed that the reason for the inability to establish a relationship between a single AMH measurement and the number of fetuses to be obtained after natural mating in this study could be individual variables, such as differences in follicular waves and follicle development, possible malfunctions in the implantation process, and the age factor.

There is widespread agreement that AMH plays a crucial role in fetal sex differentiation. AMH synthesized by Sertoli cells of human male fetus in the 7th week of pregnancy regresses the development of the Müllerian duct and the Wolffian duct forms under the influence of testosterone produced by Leydig cells (Rajpert-De Meyts et al. 1999). Pailhoux et al. (2002) reported that Sertoli cell differentiation was evidenced by the presence of AMH in goat fetuses as early as postcoital day 34. The presence of a male fetus in goats causes the Müllerian duct to begin regressing on the 46th postcoital day (Pailhoux et al. 2002). In female fetuses, AMH expression was reported to start near or soon after birth (Lutterodt et al. 2009). Despite the well-known role of AMH in intrauterine development, it is not proven to be an early biomarker that can be used to identify the male fetus, as previously claimed (Lee et al. 1996; Vigier et al. 1984). Conflicting results were obtained from a limited number of studies. Human studies concluded that maternal AMH could change (La Marca et al. 2005; Lutterodt et al. 2009) or did not change (Empey et al. 2012) according to fetal sex. Studies reporting that maternal AMH concentration was not affected by fetal sex suggested that AMH could not cross the placental barrier and was not secreted from the placenta (Lutterodt et al. 2009; La Marca et al. 2005). Nevertheless, it was proven that the placenta could express both AMH and/or receptors (AMHR2) independently from the sex in cows (Stojsin-Carter et al. 2017), humans (Novembri et al. 2015), and rats (Tata et al. 2018). There are fewer reports examining the effect of fetal sex on maternal AMH concentration during pregnancy in domestic animals (Stojsin-Carter et al. 2017; Uçmak et al. 2023). Uçmak et al. (2023) stated that the maternal AMH concentration of mares carrying a male fetus in the 4th and 5th months of pregnancy was significantly higher compared to mares carrying a female fetus; however, the difference was insufficient to determine a cut-off value. Stojsin-Carter et al. (2017) reported that the AMH level in the circulation of cows carrying a male fetus between the 35th and 135th days of pregnancy was significantly higher compared to cows carrying a female fetus, and the AMH level in the circulation of male fetuses between the 54th and 220th days of pregnancy was 1000 times higher compared to female fetuses. This study investigated the effect of fetal sex on maternal AMH in the pregnancies of goats with the potential for twining of opposite sexes and found no significant difference between the groups, unlike the two previous studies (Fig. 3, *P* > 0.05). Nevertheless, the authors argue that the increase in AMH concentration in the serum of goats carrying male fetuses until the 75th day of pregnancy, which coincides with the fetal sex differentiation period (Pailhoux et al. 2002), was remarkable, contrary to other groups, albeit having no statistical significance. Mbegbu et al. (2021) reported that the number of follicles developing in the ovaries of goats carrying twins during mid-term pregnancy was higher compared to goats carrying single offspring, and the number of primordial follicles was reduced. In the present study, pregnant goats were not grouped according to the number of fetuses carried since the effect of fetal sex on maternal AMH was the focus of the investigation. While the number of fetuses carried by pregnant goats changed ovarian follicular activity, it could also have affected maternal AMH concentration. On the other hand, individual differences in follicular development of pregnant goats, regardless of the number or sex of the fetuses, were probably the most significant cause of the high standard deviation in AMH concentration in the groups. The present study was conducted with a limited number of animals which could limit the power of statistical analysis to detect the difference among groups.

In the estrus cycle of goats, follicular waves start with an increase in serum FSH concentration (Schwarz and Wierzchoś 2000; Menchaca and Rubianes 2002). On the other hand, the FSH concentration was reported to decrease constantly throughout pregnancy in sheep (Xia et al. 2003) and goats (Kumar et al. 2015). The available data on cows and sheep indicated that low concentrations of FSH and bone morphogenetic proteins produced by oocyte and theca cells increased AMH synthesis in granulosa cells of preantral and small antral follicles (Monniaux et al. 2012). In accordance with previous literature, a measurable level of AMH concentration was detected throughout pregnancy in the current study. It was reported that maternal serum AMH concentration decreased (Nelson et al. 2010) or remained stable (La Marca et al. 2005) in pregnant women as the pregnancy progressed, and the serum AMH concentration decreased in cows near birth despite the synthesis of wave-like pattern (Stojsin-Carter et al. 2017; Monniaux et al. 2012). The present study revealed that AMH concentration continuously decreased in goats during pregnancy. This could be due to the goats with seasonal reproductive cycles entering a long anestrus period with parturition and/or the numerical reduction of follicles with a high ability to synthesize AMH in ovaries, as previously hypothesized in cows (Monniaux et al. 2012). It was concluded that future comprehensive studies are needed to reveal the maternal AMH change in goat pregnancies and the possible contributing variables.

## Conclusions

As a result, it cannot be argued that there is a relationship between maternal AMH levels and fetal sex without ensuring the uniformity of pregnant goats (age, follicular development, follicle size, number of fetuses, etc.). Similarly, a single AMH measurement during estrus synchronization does not indicate the number of offspring to be obtained after natural mating. On the other hand, to the authors’ knowledge this study is the first to investigate the level of AMH in pregnant goats and its progression depending on fetal sex. Since the predictions about litter size in sheep and goats provide significant data in field conditions, it is thought that the study pave the way on further research by ensuring repeated AMH measurement and standardizing individual differences in large sample groups.

## Data Availability

The data that support the findings of this study are available from the corresponding author upon reasonable request.
